# Migraine as a risk factor for retinal vascular events and maculopathies: a systematic review and meta-analysis of 47 million individuals

**DOI:** 10.1186/s10194-026-02353-8

**Published:** 2026-04-15

**Authors:** Mohamed I. Mohamed, Masa I. Halwag, Nadeen H. Mahmoud, Michael Salib, Mona AF Nada

**Affiliations:** 1 Faculty of Medicine, Alexandria University, Champollion street, Al Mesallah Sharq, Alexandria, Al Attarin, Alexandria Governorate 5372066 Egypt; 2 Research Support Division, Alexandria Students’ Scientific Association, Alexandria University, Faculty of Medicine, Alexandria, Egypt; 3 Department of Neurology, Faculty of Medicine, Cairo University, Cairo, Egypt

**Keywords:** Migraine, Retinal artery occlusion, Retinal vein occlusion, Retinal stroke, Maculopathy, Macular degeneration, Central serous chorioretinopathy, Systematic review, Meta-analysis

## Abstract

**Background:**

Migraine is increasingly recognized as a systemic disorder associated with cerebrovascular disease, endothelial dysfunction, and microvascular retinal changes. Given shared features between the retina and cerebral circulation, retinal vascular events (RVEs) and maculopathies may represent additional vascular manifestations of migraine. We aim to meta-analyze evidence on RVE and maculopathy risk in migraine.

**Methods:**

We conducted a PRISMA-compliant systematic review and meta-analysis (PROSPERO: CRD420251250431). Web of Science, PubMed, Scopus, and meeting abstracts were searched through January 2026, without language or date restrictions. Observational studies comparing the risk of RVEs or maculopathies in migraineurs versus non-migraineurs were included. Random-effects meta-analyses were performed using pooled risk ratios (RRs) from best adjusted analyses with 95% confidence intervals (CIs). Study quality was assessed using the Newcastle–Ottawa Scale.

**Results:**

Thirteen studies encompassing 47,042,175 individuals met the inclusion criteria. An adjusted analysis suggested migraine was associated with a significantly increased risk of any RVE (RR 2.04, 95% CI 1.53–2.72). Migraineurs demonstrated elevated risks of any RAO (RR 2.12, 95% CI 1.25–3.60), central RAO (RR 1.62, 95% CI 1.14–2.30), and branch RAO (RR 1.94, 95% CI 1.57–2.40). Similarly, the risk of any RVO was increased (RR 1.72, 95% CI 1.44–2.04), including central and branch forms. Migraine with aura conferred a higher arterial occlusion risk compared to migraine without aura in two studies. Evidence on the protective effects of therapies remains inconclusive. Three studies suggest associations with neovascular age-related macular degeneration and central serous chorioretinopathy. Ten studies were rated as good according to the AHRQ standards.

**Conclusions:**

Migraine is associated with a significantly increased risk of retinal arterial and venous occlusive events, yet current literature is limited by miscoding from administrative database studies. Evidence on increased maculopathy risk in migraine remains elusive. These results support heightened awareness of retinal vascular complications in migraineurs and proactive ocular monitoring, particularly those with aura. Past links implying higher stroke risk following RAO, combined with migraine’s established association with stroke, hint at the need for primary stroke prevention in migraineurs with RAO. Prospective studies to clarify causality and the impact of migraine therapies - particularly anti-CGRPs - on retinal vascular risk are warranted.

**Graphical Abstract:**

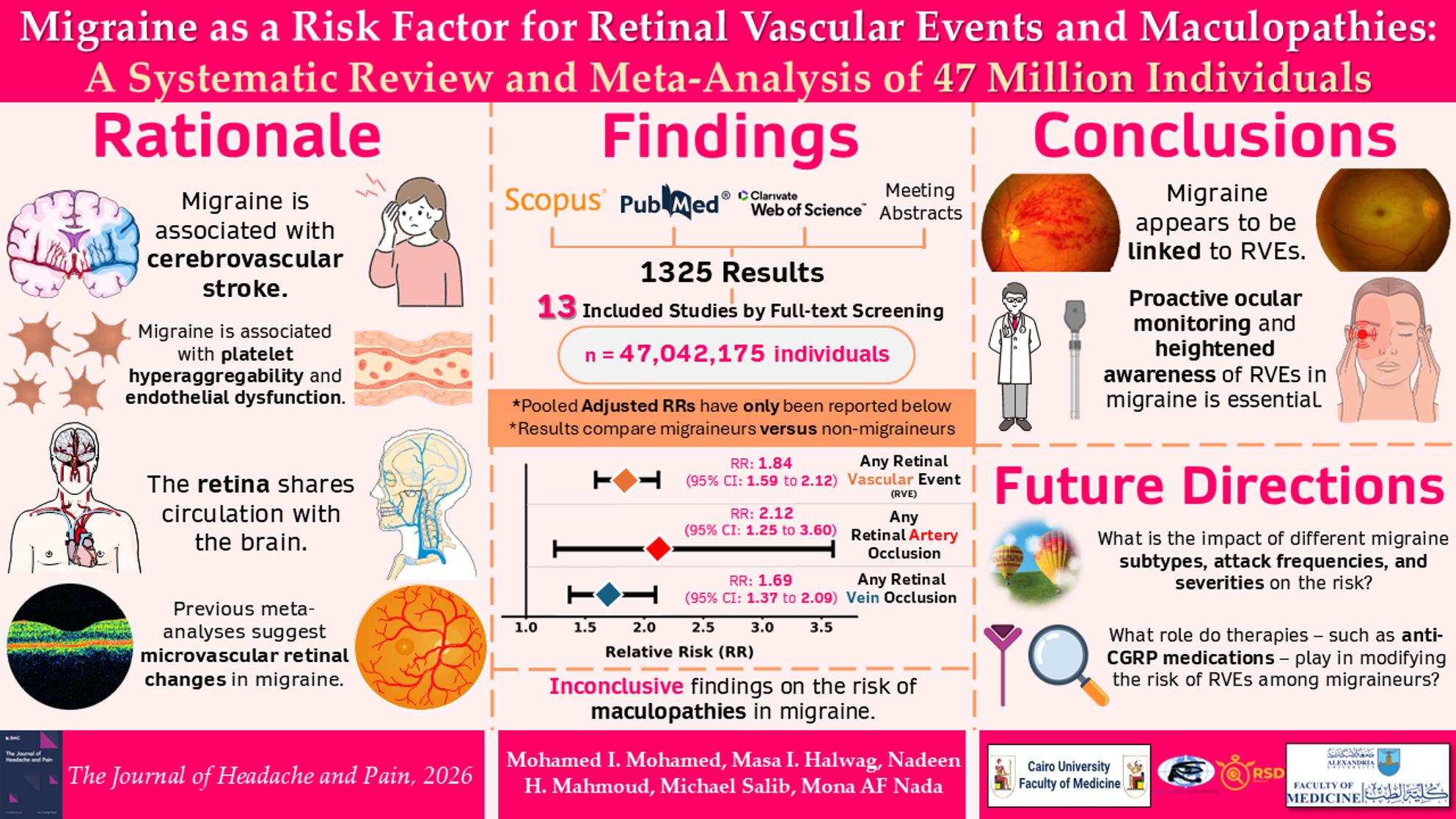

**Supplementary Information:**

The online version contains supplementary material available at 10.1186/s10194-026-02353-8.

## Introduction

Migraine is one of the most common neurological disorders affecting over a billion people worldwide and is considered a cause of significant disability globally [1]. Although conceptualized primarily as a headache disorder, migraine is increasingly understood as a neurovascular condition involving complex interactions between neuronal excitability, vascular dysregulation, inflammation, and endothelial dysfunction [2, 3]. Supporting this is evidence from the trigeminovascular hypothesis [4] and from the established efficacy of monoclonal antibodies targeting calcitonin gene-related peptide in adults [5]. Within this context, migraine has been increasingly studied in relation to vascular disease and is recognized as a risk factor for systemic and cerebrovascular diseases, including ischemic stroke [6].

Migraine-related vascular dysfunction is widespread and is not confined to the cerebral circulation; this finding has been consistent in the literature. For example, migraine has been associated with the involvement of the coronary microvasculature and the subsequent increased risk of ischaemic heart disease in both women [7] and men [8]. Vascular dysfunction in migraine may even extend to the dermal microcirculation, as demonstrated by Tietjen et al., who reported an association between migraine and livedo reticularis, supporting involvement of cutaneous microvasculature [9]. Given this widespread vascular involvement observed in migraine, it is plausible that retinal vessels are similarly affected, particularly as the retina shares embryologic and physiologic characteristics with the cerebral circulation [10]. Supporting this concept, retinal microangiopathic changes have been consistently reported in migraine [11, 12], and migraine with aura (MwA) has been associated with foveal avascular zone enlargement and reduced retinal perfusion density in a 2023 meta-analysis of 16 studies [11].

These microvascular alterations may provide a biological basis for the occurrence of clinically overt retinal vascular events (RVEs). RVEs comprise disorders characterized by impaired retinal perfusion, including central and branch retinal artery occlusion (CRAO and BRAO, respectively), in addition to central and branch retinal vein occlusion (CRVO and BRVO, respectively). These events can be debilitating and have been associated with an increased risk of mortality [13], and RVO has been shown to cause substantial deterioration in vision-related quality of life [14].

Given the clinical and functional burden associated with these conditions, considerable interest has emerged in identifying their potential risk factors, including migraine. However, there has been greater variability in results while examining the literature. For instance, Battagliola et al. did not find a statistically significant association between migraine and any retinal vascular event [15], while Al-Moujahed et al. found a statistically significant association between migraine and any RAO - reaching up to 248% compared to non-migraineurs [16]. Not to mention, Tilleul et al. found a statistically significant association between migraine and any RVO, twice that of those without migraine [17]. This heterogeneity in reported outcomes underscores the need for a systematic review and meta-analysis to elucidate the association between migraine, RVEs, and maculopathies and to provide robust risk quantification.

Accordingly, our study purports to investigate the association between migraine and RVEs, including both arterial and venous occlusions, as well as for maculopathies. Furthermore, we hope to explore whether current migraine therapeutic options may influence the risk of those events.

## Methods

Our study was conducted following the updated Preferred Reporting Items for Systematic Reviews and Meta-analyses (PRISMA) guidelines [18]. Further, we prospectively registered the protocol of the study in PROSPERO (ID: CRD420251250431).

### Search strategy

A comprehensive search strategy was conducted across three databases (MEDLINE, PubMed, and Scopus) on the 11th of December, 2025. Later on, an updated query was conducted on the 25th of January, 2026. We also supplemented this search using preprints from the Scopus database, in addition to querying annual meeting abstracts, namely those from the Association of Research in Vision and Ophthalmology, the American Academy of Neurology, the European Academy of Neurology, the International Headache Society, the North-American Neuro-ophthalmology Society, and the American Academy of Ophthalmology. The strategy included the use of Medical Subject Headings (MeSH) terms, and no restrictions related to linguistics or publication period were applied. After identifying relevant articles, we reviewed the bibliographies of the included studies to ensure no evidence was missing. Some of the search terms used were migraine, retinal artery occlusion, retinal vein occlusion, and transient vision loss. For the full search strategy utilized, refer to Additional File 1.

### Screening and study selection

The platform used for the screening process was Covidence [19]. Two independent authors screened the studies based on our eligibility criteria below. Conflicts were resolved by a third author. The screening process was conducted in two stages: title and abstract, followed by full-text screening.

### Inclusion criteria

We included studies that assessed the risk of RVE, including: (retinal artery occlusion - branch or central, retinal vein occlusion - branch or central, and amaurosis fugax) and maculopathies (such as neovascular age-related macular degeneration (AMD), paracentral acute middle maculopathy (PAMM), or central serous chorioretinopathy (CSCR)), in migraine patients (with or without aura) diagnosed using established criteria (such as the ICHD-3 criteria [20]) versus healthy controls. Included study designs were cohorts and case-control studies. We also included annual meeting abstracts to ensure comprehensiveness, following the guidance provided in a previous commentary by Scherer et al. [21]. Diligent attempts were made to reach out to authors of relevant abstracts to enquire about their full-text status.

### Exclusion criteria

Studies that assessed microvascular changes or retinal nerve fiber thickness were excluded. In case we encountered two or more studies on the same population from the same dataset, we resorted to excluding the studies with the smallest sample size, ensuring the independence of each study sample and no population overlap. We also excluded case reports, reviews, meta-analyses, animal-based studies, and commentaries.

### Data extraction

Relevant data were extracted into a Google extraction spreadsheet. Each author extracted data from a given number of studies. A second author reviewed the extracted data to ensure accuracy. Primary outcomes of interest were the risk of any RVE, RAO, RVO, and any maculopathy. Secondary outcomes of interest were the risk of CRAO, BRAO, CRVO, BRVO, and a comparison of RVE risk in MwA versus without aura (MwoA). These outcomes (whenever available) were extracted in the form of relative risk (RR) and 95% confidence intervals (CI) in migraineurs versus non-migraineurs. Supplementary files were also inspected to ensure no relevant data were left out. The baseline characteristics table included information on the study design, country, data collection period, participating centre(s) and/or databases, total sample size, age and sex distribution, proportion of smokers, and a brief conclusion of each study’s findings.

### Quality assessment

We assessed the quality of the included studies using the Newcastle-Ottawa scale (NOS) [22]. Similar to the screening process, this step was conducted by two independent quality assessors. This scale evaluates studies based on three domains: selection of the study groups, comparability, and outcomes. NOS scores range from zero to nine, with scores greater than seven indicating better methodological quality.

According to AHRQ quality assessment standards (maximum of 9 stars), studies are categorized as good, fair, or poor quality based on domain-specific scores. Good-quality studies receive 3–4 stars for selection, 1–2 stars for comparability, and 2–3 stars for outcome/exposure. Fair-quality studies must achieve at least 2 stars in selection, 1–2 in comparability, and 2–3 in outcome/exposure. Poor-quality studies are characterized by only 0–1 star in selection, no stars in comparability, and 0–1 star in outcome/exposure.

### Statistical analysis

We conducted a meta-analytic synthesis of the risk of RVEs using a random-effects model from the meta package on R software version 4.4.1 [23]. The metagen function pooled risk ratios (RRs) and 95% CIs. Studies with narrower (more precise) CIs received a greater weight in the final effect estimate calculation. Whenever we encountered a study with multiple different RRs (for example, unadjusted model, univariate adjusted model, multivariate adjusted model), we decided to preferentially extract the effect estimate that has been adjusted to the greatest number of confounding variables. Of note, studies only reporting unadjusted RRs were still included in the primary analysis, albeit a dedicated subgroup analysis for adjusted and non-adjusted studies was planned, effectively helping distinguish the contribution of migraine to the risk compared to other confounders. This approach maintains inclusivity of the broader literature as well as provides insights into the effect of adjusting confounders on RVE risk, without compromising on statistical power. Heterogeneity was reported using the I^2^ statistic, which is an inconsistency measure developed by Higgins et al. [24], allowing the quantification of between-study inconsistency on a percentage scale from 0% to 100%. I^2^ statistic values of 25%, 50%, and 75% were categorised as low, moderate, and high heterogeneity scores, respectively. A leave-one-out test allowed us to identify single-study sources of heterogeneity. Further, we carried out a sensitivity analysis to examine the effect of omitting studies that were only published in abstract form, guided by Scherer et al. [21] Whenever a study reported odds ratios [25](ORs) or hazard ratios [26](HRs), we utilized established methods for conversion and estimation of the RR from OR/HR. Because RVEs are relatively rare outcomes in the studied populations, the use of established statistical methods to estimate and infer RRs from ORs or HRs was considered reasonable, especially given how both methods were extensively utilized in the Neurology/Ophthalmology literature (OR to RR [27–29] and HR to RR [27, 30, 31]).

## Results

### Screening outcome

**Fig. 1:**
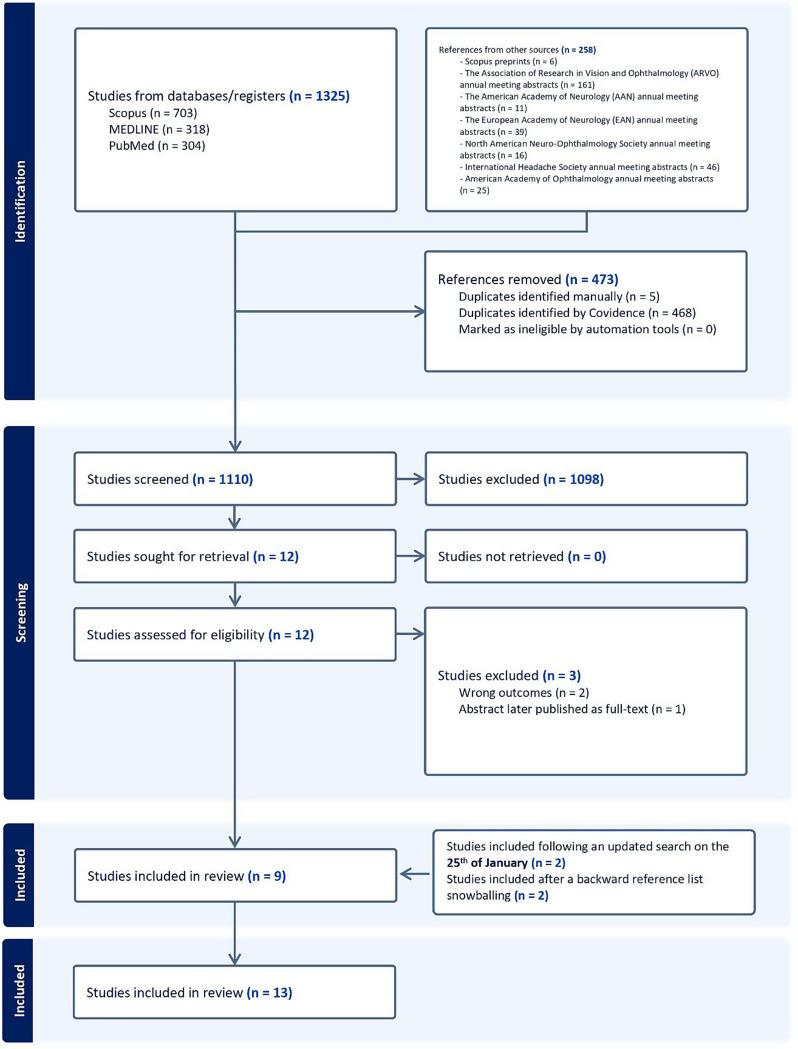
PRISMA Flowchart

Of 1583 potentially relevant articles and abstracts, after removing duplicate entries (473), 1110 studies were screened. Nine studies eventually met our eligibility criteria, with four identified following an updated search and a backward reference snowballing technique, making the total number 13 [15–17 ,32–41. (Fig. 1). Those studies which were excluded by full-text screening have had their exclusion reasons summarized in Additional File 1 (Table S1).

### Baseline characteristics

The identified studies in this systematic review were mainly observational multicentric retrospective cohorts [16, 32, 34–36, 38] or case-control studies [15, 33, 37, 39, 40], with three prospective ones [17, 37, 41]. Gil et al. [38] was the only eligible meeting abstract to be included in this review. The total sample size was 47,042,175 individuals. Ten studies reported the risk of retinal vascular events [15–17, 32, 34–36, 38, 39, 41]. Three studies investigated the contribution of migraine towards the risk of CSCR [32, 37, 40]. Kuang et al. [33], in their Taiwanese retrospective cohort, evaluated migraine as a potential risk factor for neovascular AMD. Among studies assessing the risk of retinal vascular events, Ho et al. [36] assessed both retinal arterial and retinal venous occlusion risks. Four studies focused on RVO (central or branch) [15, 17, 38, 41], while four other studies calculated the risk of RAO only (central or branch) [16, 34, 35, 39]. Mean ages typically clustered in the 40s, with ranges between 28.45 [32] and 71.4 [33]. Sex distribution was highly variable among studies, with male percentages ranging from 18.9% [16] to 60.4% [33]. Six of the 13 studies were conducted in the United States of America [16, 34, 35, 38, 39, 41], with the rest involving Europe (Italy [15] and France [17]) or Asia (Taiwan, India, and Israel) [32, 33, 36, 40]. One study involved centers from four countries (Lebanon, the United States, Brazil, and Egypt). [37] An in-depth breakdown of the study and patient characteristics is illustrated in Table 1.

**Table 1:** Baseline characteristics of the included studies

Author, Year	Study Design	Country and data collection period	Follow-up Duration in years – mean (SD)	Participating centre(s) and/or databases	Total sample size	Migraine characteristics	Age and Sex Distribution	% Smokers	Brief Conclusion
Al-Moujahed 2021	Multicentric, retrospective, cohort.	USA, 2007–2016	2.89 (1.8) in the migraine group. 3.93 (1.8) in the no-migraine group.	IBM MarketScan Commercial Claims and Encounters database	Migraine: 418, 965 No Migraine: 418, 965 RAO and Migraine: 1060 (0.25%) RAO and no Migraine: 335 (0.08%)	N/A	Age in mean (SD): Migraine Age: 41.2 (11.9) No Migraine Age: 41.2 (11.9) Sex distribution in number (percent): Males with migraine: 79,275 (18.9%) Males without migraine: 79,275 (18.9%)	Migraine: 33,243 (7.9%) No Migraine: 17,123 (4.1%)	Migraine is linked to a higher risk of all forms of RAO, with migraine accompanied by aura conferring a greater risk than migraine without aura.
Battagliola 2021	Multicentric, case-control study.	Italy	N/A	Two Italian hospitals from central and northern Italy.	Cases with CRVO: 203 Control group without CRVO: 339	N/A	Age in mean (SD): Cases with CRVO: 68.16 (9.013) Control group without CRVO: 55.64 (11.77) Sex distribution in % Male cases with CRVO: 47.29 Male Control group without CRVO: 51.03%	Cases with CRVO: 41.37% Control group without CRVO: 21.24%	Migraine is not significantly linked to greater odds of CRVO.
Gil 2025	Multicentric, retrospective, cohort. (An ARVO annual meeting abstract)	USA	N/A	TriNetX	Migraine: 1,635,632 No migraine: 1,635,632	N/A	N/A (Propensity score matching baseline characteristics such as age, gender, and race)	N/A	Individuals younger than 55 years with a history of migraine have an almost twofold increased risk of developing RVO compared with those without migraine.
Ho 2024	A multicentric, nationwide, retrospective cohort.	Taiwan	7.09 (3.37)	Taiwan National Health Insurance Research Database	Migraine: 628,760 No migraine: 628,760	9% MwA 29.94 MwoA 61.07% other subtypes	Age in mean (SD): 45.35 (14.86) Sex distribution in %: 27.07% males	15,532 (1.24%)	Migraine is associated with an increased risk of RAO and RVO, with the greatest RAO risk in MwA. NSAIDs, propranolol, and flunarizine may reduce this risk among migraine patients.
Klein 2008	Population-based prospective cohort.	USA, 15-year follow-up. The study continued from 1987 to 2005.	At least 5 years of follow-up, but up to 15 years.	A private census of the population of Beaver Dam.	Total: 4068 14.9% with Migraine: 606	N/A	43.1% males Individuals aged 43 to 84	19.4% active smokers.	Migraine history was significantly associated with BRVO, but not CRVO.
Kuang 2022	A nationwide population-based, multicentric case-control study.	Taiwan, January 2010 and December 2016	N/A	Taiwan National Health Insurance Research Database.	Total: 101,665 Patients with neovascular AMD: 20,333 (6.1% with migraine) Controls without neovascular AMD: 81,332 (4.9% with migraine)	N/A	Mean age: 71.4 years Males’ percentage in patients with neovascular AMD: 60.4% Males’ percentage in controls: 59.5%	N/A	The study offers population-based evidence that persons with migraine have 20% higher risk of subsequently being diagnosed with neovascular AMD.
Lusk 2024	Retrospective, multicenter cohort.	USA, 2005–2015	At least 2 years	State Inpatient Databases and State Emergency Department Databases from New York (2006–2015), California (2003–2011), and Florida (2006–2015)	Total: (39,835,024) Patients with hospital-ascertained migraine: 1,109,140 Patients without hospital-ascertained migraine: 38,725,884	N/A	Age in mean (SD): 46.7 (19.7) Males: 17,631,626 (44.6%)	5,911,986 (14.8)	No definite association between migraine and CRAO.
Tilleul 2011	A prospective, single-center questionnaire-based case-control study.	France, January - October 2009	N/A	Ophthalmology Department of the Créteil Intercommunal Hospital, University of Paris-XII-Henri-Mondor.	Total:179 61 patients with RVO, 118 controls. 24 with migraine (13.3%)	N/A	Age in mean (SD): 67.4 (8.7) 57% males.	27.8%	Migraine is significantly associated with CRVO and BRVO.
Uppuluri 2021	Retrospective, multicentre case-control study.	USA, 2002–2014	N/A	National Inpatient Sample Database	Total: 5952 Cases with CRAO: 522 (6.7% with migraine) Controls: 5430 (2.7% with migraine)	N/A	Mean age: 37.3 58.4% males in the control group. 56.6% males in the case group.	23.7% smokers in the control group. 32.3% smokers in the case group.	Migraine was significantly associated with CRAO.
Lusk 2026	Retrospective, multicentre cohort study.	USA, 2018–2023	At least 12 months	Computerized files of Marketscan by Merative, a proprietary health insurance program	Total: 900,370 (58,679 using a CGRP inhibitor, 841,691 not using a CGRP inhibitor)	N/A	Mean (SD) for age: 41 (14.8) 22.2% males.	N/A	In individuals with migraine, the initiation of anti-CGRP therapy did not significantly increase the risk of CRAO.
Nitzan 2026	Retrospective, multicentre cohort study.	Israel, Follow-up for 5 years from the Index date.	5 years.	TriNetX	Total: 827,326 (413,663 with migraine and 413,663 without migraine)	N/A	Mean (SD) for age: 28.45 (6.8) 19.2% males.	4% with nicotine dependence. No data on smokers.	Migraine was associated with a significantly increased risk of incident central serous choroioretinopathy in a large, real-world cohort.
Mansour 2017	Prospective, multicentre case control study.	Lebanon, Brazil, Egypt, and the United States. January 2015 to February 2016		Six different university-based centers from four countries.	Total: 166 (83 CSCR, and 83 controls)		Mean (SD) for age: Control: 45.9 (15.7) CSCR: 46 (12.2) 80.7% males	41% smokers in the control group, and 50.6% in the CSCR group.	Migraine was strongly associated with CSCR (*p* = 0.01).
Kumawat 2021	Case-control study	India. July 2017 to December 2018.	N/A	A tertiary eye care center in North India.	Total: 169 Cases: 87 Controls: 82	N/A	Mean (SD) for age: Control: 35.7 (10.8) Cases: 36.9 (7.8) M: F ratio: Controls: 2.9:1 Cases: 4.8:1	21.9% in the control group. 17.2% in cases.	No significant association between migraine and CSCR.

**Abbreviations: USA**: United States of America, **IBM**: International Business Machines, **RAO**: Retinal Artery Occlusion, **CRAO**: Central Retinal Artery Occlusion, **CRVO**: Central Retinal Vein Occlusion, **RVO**: Retinal Vein Occlusion, **BRVO**: Branch Retinal Vein Occlusion, **ARVO**: The Association for Research in Vision and Ophthalmology, **NSAIDs**: Non-steroidal anti-inflammatory drugs, **AMD**: Age-related Macular Degeneration, **CGRP**: Calcitonin Gene-Related Peptide, **CSCR**: Central Serous Choroidoretinopathy, **MwA**: Migraine with Aura, **MwoA**: Migraine without Aura

**M: F ratio**: Male to female ratio

### Quality assessment

Overall, the methodological quality of the included studies was variable but generally moderate to high. Using the AHRQ-standardized assessment, ten of the twelve studies were rated as good quality, with total scores ranging from 7 to 9 out of 9. These studies were predominantly large population-based cohort or well-designed case-control studies, with robust exposure ascertainment, appropriate selection of comparison groups, and adequate outcome assessment.

Two small hospital-based case-control studies were rated as poor quality [15, 17], primarily due to limited representativeness, insufficient comparability between cases and controls, and lack of adjustment for key confounding variables.

One eligible study was a conference abstract [38], which lacked key comprehensive methodological details. Consequently, this study was considered to have an unclear risk of bias. Table 2 demonstrates the details for each study.

**Table 2:** Quality assessment of the included studies

Cohort Studies										
Author, Year	SELECTION				COMPARABILITY	OUTCOMES			Total points/9	AHRQ Standardised Rating
	1) Representativeness of the exposed cohort	2) Selection of the non-exposed cohort	3) Ascertainment of exposure	4) Demonstration that outcome of interest was not present at start of study	1) Comparability of cohorts on the basis of the design or analysis	1) Assessment of outcome	2) Was follow-up long enough for outcomes to occur	3) Adequacy of follow-up of cohorts		
Al-Moujahed 2021	*	*	*	*	**	*		*	**8**	**Good**
Ho 2024	*	*	*	*	**	*	*	*	**9**	**Good**
Klein 2008	*	*	*	*	**	*	*	*	**9**	**Good**
Lusk 2026	*	*	*	*	**	*		*	**8**	**Good**
Lusk 2024	*	*	*	*	**	*	*	*	**9**	**Good**
Nitzan 2026	*	*	*	*	*	*	*	*	**9**	**Good**
**Case-Control Studies**										
**Author**,** Year**	**SELECTION**				**COMPARABILITY**	**EXPOSURE**			**Total points/9**	**AHRQ Standardised Rating**
	Is the Case Definition Adequate?	Representativeness of the Cases	Selection of Controls	Definition of Controls	Comparability of Cases and Controls on the Basis of the Design or Analysis	Ascertainment of Exposure	Same method of ascertainment for cases and controls	Non-response rate		
Battagliola 2021		*			*	*	*		**4**	**Poor**
Kuang 2022	*	*	*	*	**	*	*	*	**9**	**Good**
Tilleul 2011		*			*		*	*	**4**	**Poor**
Uppuluri 2021	*	*		*	**	*		*	**7**	**Good**
Mansour 2017	*		*	*	**	*	*	*	**8**	**Good**
Kumawat 2021	*	*	*	*	**	*		*	**8**	**Good**

## Meta-analysis

### Risk of retinal vascular events in migraine

**Fig. 2:**
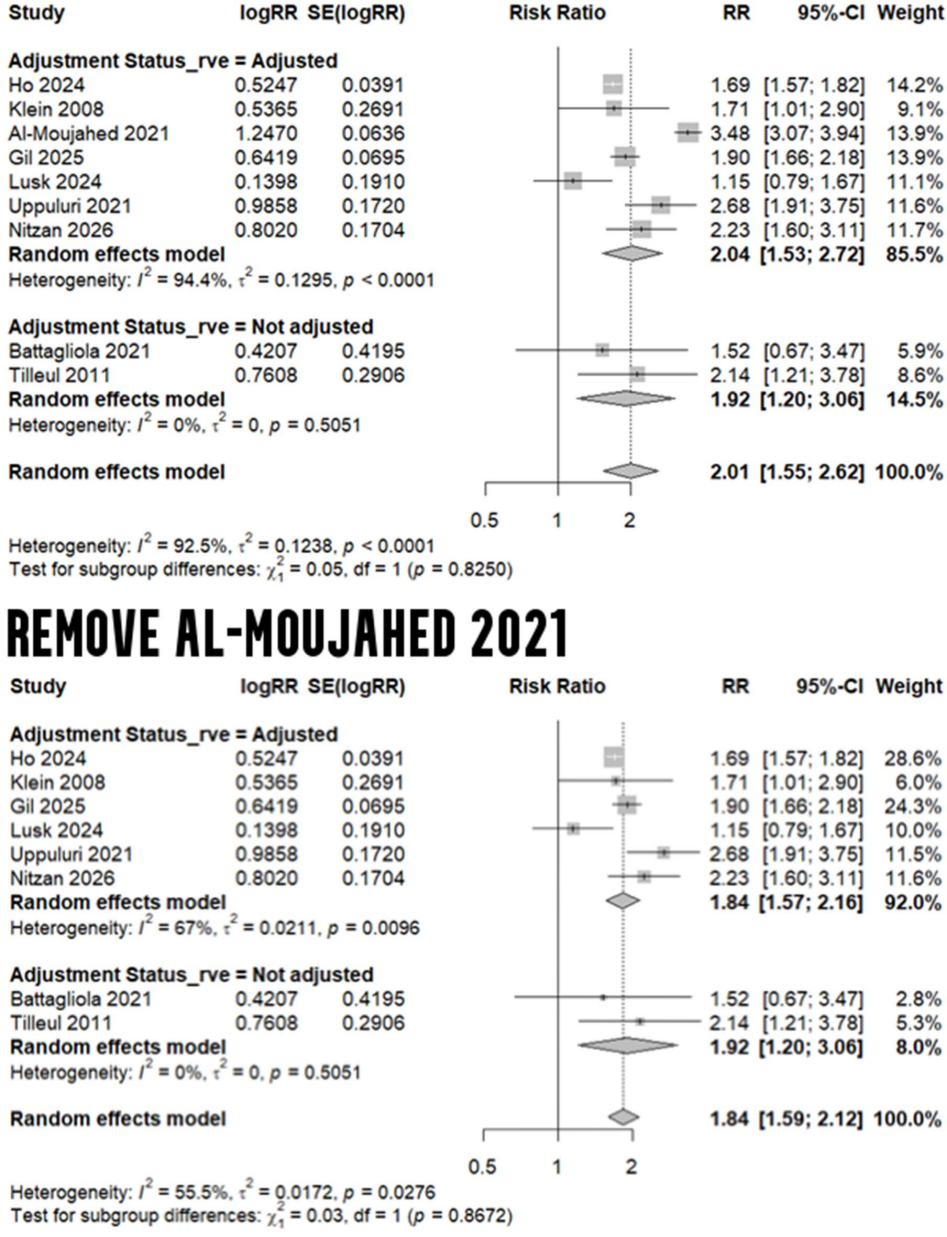
Risk of RVE in migraine

In the primary meta-analysis of 9 studies [15–17, 32, 35, 36, 38, 39, 41] reporting on the risk of any retinal vascular event (RAO or RVO) in migraineurs versus non-migraineurs, we revealed an elevated risk (RR: 2.01, 95% CI: 1.55 to 2.62, I^2^: 92.5%). While we focused on obtaining the best-adjusted RRs from multivariate models in the included studies during extraction, 2 studies by Battagliola et al. [15] and Tilleul et al. [17] reported unadjusted estimates only. Therefore, to demonstrate the findings from studies with adjusted-only models, we conducted a separate analysis of 7 studies [16, 32, 35, 36, 38, 39, 41] reporting adjusted risk ratios only; this indicated a similar result (RR: 2.04, 95% CI: 1.53 to 2.72, I^2^: 94.4%). Given the high heterogeneity, we also conducted a leave-one-out test to omit the outlier (Al-Moujahed et al. [16]), yielding a statistically significant result with lower heterogeneity (RR: 1.84, 95% CI: 1.59 to 2.12, I^2^: 55.5%) - Fig. 2. Further, results of a sensitivity analysis to examine the effect of removing Gil et al. [38]- the meeting abstract with unclear risk of bias - is shown in Additional File 1 (FigureS1).

Table 3 provides a breakdown of the risks of RVO and RAO in the literature, alongside a list of adjusted confounders. Table S2 from Additional File 1 summarizes the effect measures reported in each study.

### Risk of retinal arterial occlusion in migraine

**Fig. 3:**
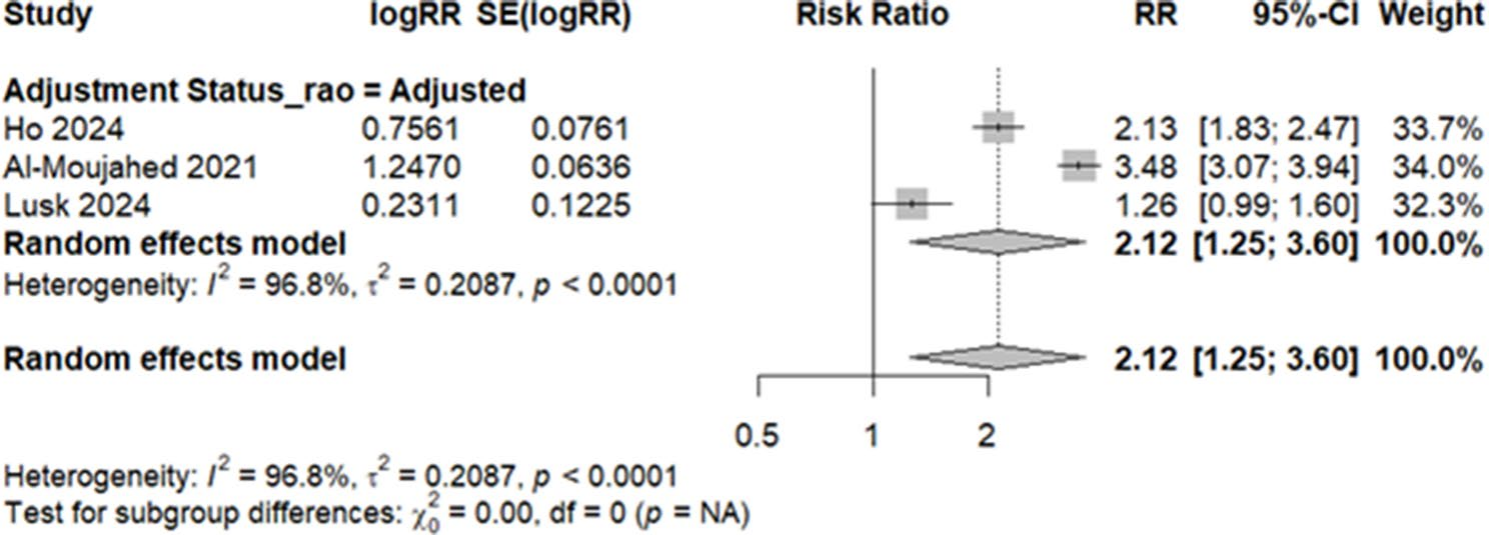
Risk of any RAO in migraine

**Fig. 4:**
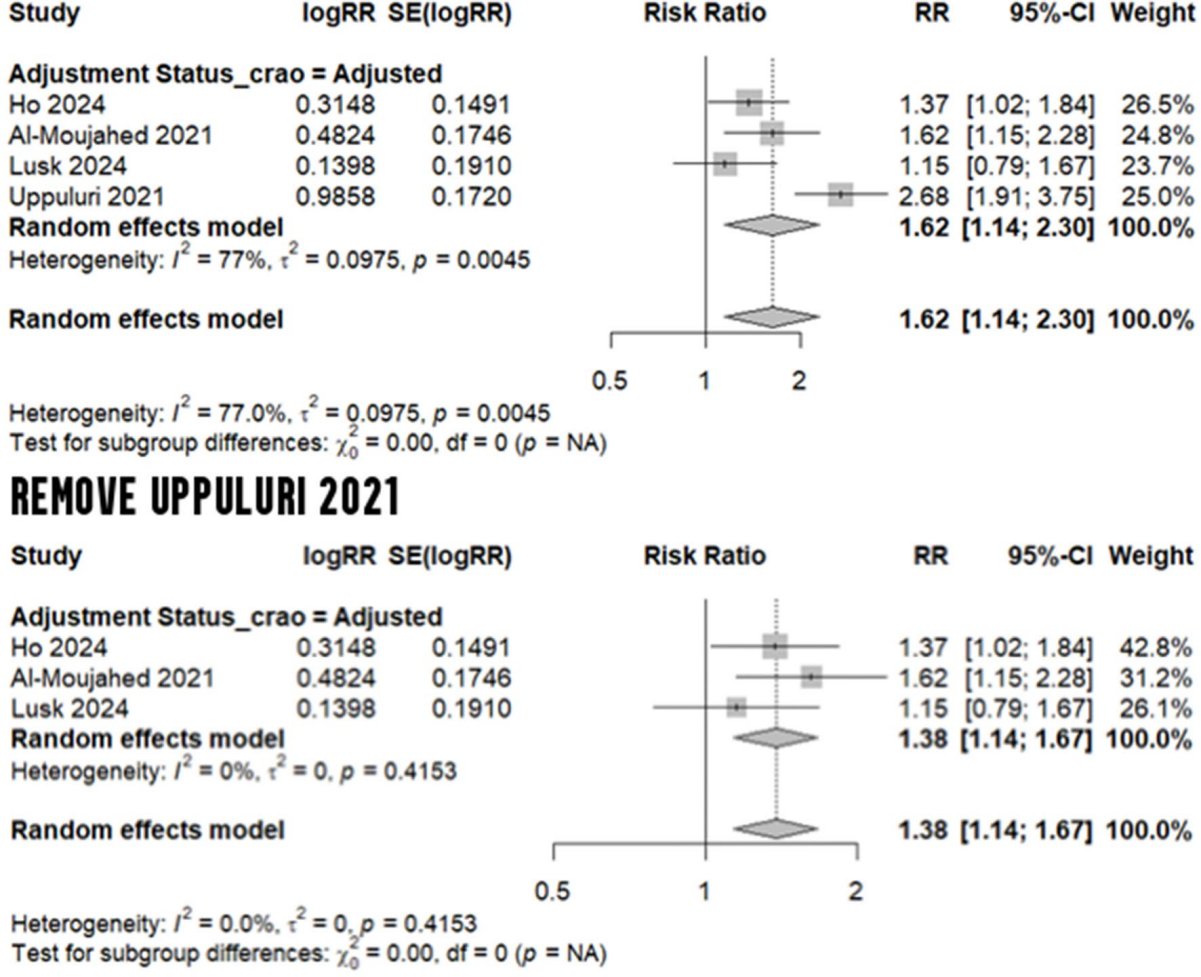
Risk of CRAO in migraine

**Fig. 5:**
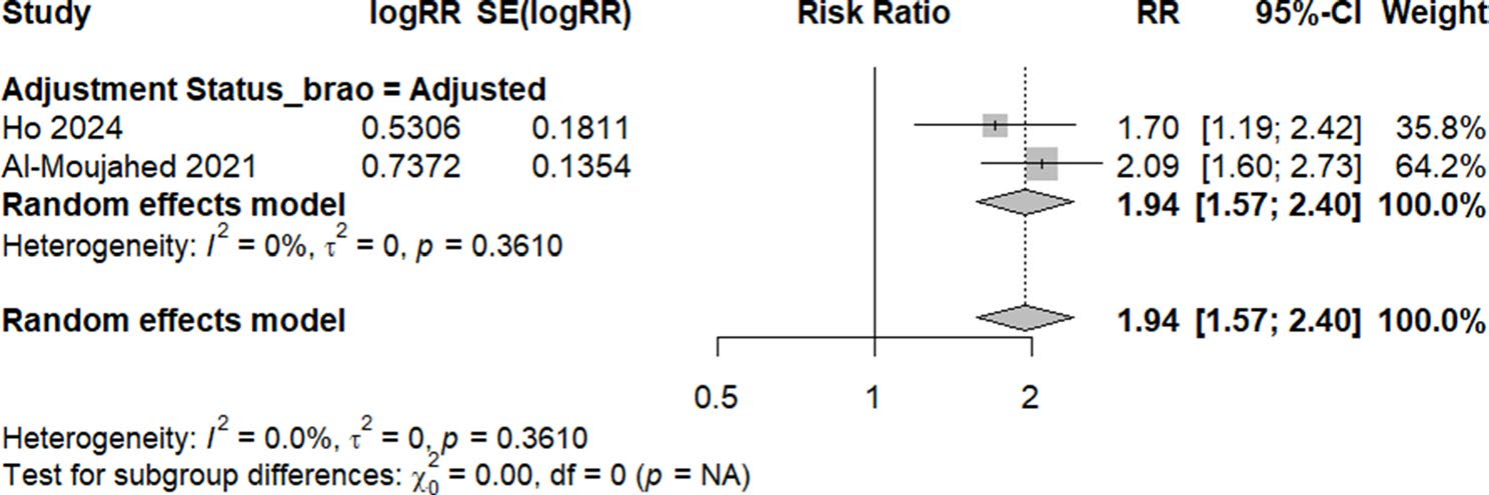
Risk of BRAO in migraine

Three studies - with adjusted risk ratios - compared the risk of any RAO (including central, branch, transient, or others) in migraineurs versus non-migraineurs [16, 35, 36]. Upon conducting a meta-analysis, we calculated an elevated risk of any RAO (RR: 2.12, 95% CI: 1.25 to 3.60, I^2^: 96.8%), albeit with high heterogeneity (Fig. 3). A meta-analysis of four studies [16, 35, 36, 39] reporting the adjusted risk for CRAO estimated an increased risk by 62% in migraineurs (RR: 1.62, 95% CI: 1.14 to 2.30, I^2^: 77%). Following a leave-one-out test, which removed Uppuluri et al. [39], a 38% increased risk of CRAO was estimated without any heterogeneity (RR: 1.38, 95% CI: 1.14 to 1.67, I^2^: 0%) - Fig. 4. With regard to BRAO, we meta-analyzed the two studies with adjusted estimates [16, 36], yielding a 94% elevation in risk for migraineurs (RR: 1.94, 95% CI: 1.57 to 2.40, I^2^: 0%) - Fig. 5.

### Risk of retinal venous occlusion in migraine

**Fig. 6:**
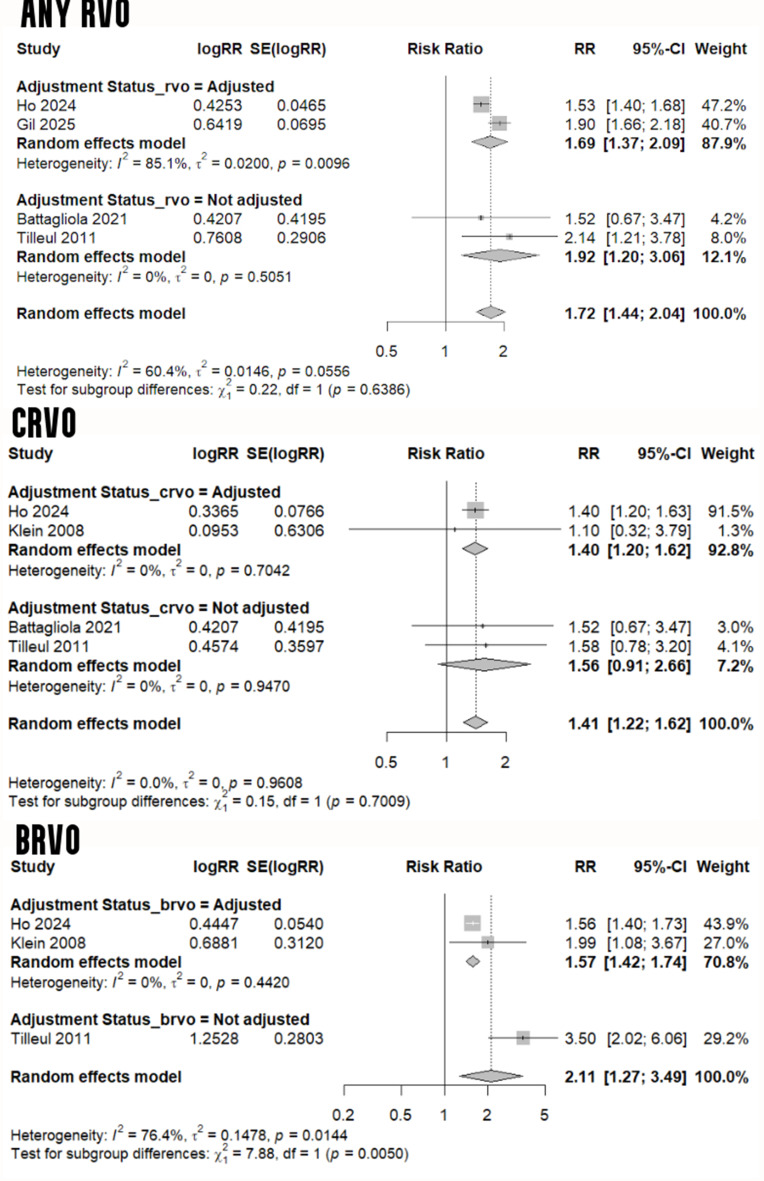
Risk of RVO in migraine

Migraine was associated with a greater risk of any RVO, as highlighted in our meta-analysis of four studies [15, 17, 36, 38] (RR: 1.72, 95% CI: 1.44 to 2.04, I^2^: 60.4%). This increased risk was also established in a separate analysis of studies with adjusted risk ratios [36, 38] (RR: 1.69, 95% CI: 1.37 to 2.09, I^2^: 85.1%). Furthermore, in an analysis of four studies [15, 17, 36, 41] comparing the risk of CRVO in migraineurs versus non-migraineurs, a 41% increased risk (95% CI: 1.22 to 1.62) was noticed with 0% heterogeneity. Similarly, the adjusted risk ratio for BRVO reached 1.57 in migraineurs based on a meta-analysis of two studies [36, 41] (95% CI: 1.42 to 1.74, I^2^: 0%) - Fig. 6. Results of a sensitivity analysis to examine the effect of removing Gil et al. [38] - the meeting abstract with unclear risk of bias - is shown in Additional File 1 (Figure S2).

### Impact of covariates (migraine subtype, age, gender, and treatment) on the risk

Al-Moujahed et al. [16] and Ho et al. [36] compared the risk of RAO in individuals having MwA versus those with MwoA; a meta-analysis of these two studies indicated a 57% increased risk of RAO in individuals having MwA compared to those with MwoA (Figure S3 in Additional File 1). Further, while both studies agreed that the increased RAO risk extends to all age groups, a disparity was clear over which age group is at the greatest risk. Al-Moujahed et al. [16] reported that those aged > 50 have a greater RAO risk compared to individuals with migraine < 50 (HR 2.205 [95% CI 1.961–2.478]; *P* < 0.0001). Ho et al. [36], on the other hand, identified individuals aged 20–40 and those aged ≥ 80 as bearing the greatest RAO risk. As for treatments and their impact on the risk of RAO, when comparing migraine patients with versus without medications, only NSAIDs, propranolol, and valproate were found to reduce the risk (aHR: 0.18, 0.61, 0.55, respectively). Triptans, ergotamine, metoprolol, topiramate, and flunarizine had no significant impact on the risk of any RVE. In a 2026 study by Lusk et al. [34], anti-CGRP therapies - whether for abortive or prophylactic use - did not significantly increase the risk of CRAO.

### Risk of maculopathies in migraine

One case-control study by Kuang et al. [33] identified migraine as an independent risk factor for neovascular AMD. In their multiple logistic regression analysis of 5184 individuals, after adjusting for age, sex, monthly income, geographic location, residential urbanization level, hyperlipidemia, diabetes, coronary heart disease, hypertension, and previous cataract surgery, migraine cases had a 20% elevated odds - aOR: 1.201 (95% CI 1.123 ~ 1.284; *p* < 0.001). In another TriNetX-based propensity-score matched retrospective cohort involving 827,326 individuals, those with migraine had a 174% increased risk of CSCR (aHR: 2.74, 95% CI: 1.66 to 4.50). This risk persisted among sex-stratified, extended follow-up, and aura/without aura analyses [32]. A prospective, univariate analysis by Mansour et al., involving an intercontinental sample of 166 cases and controls, identified migraine as a strong risk factor for CSCR, where 18.5% of cases had migraine, compared to only 6% in the control group [37]. In another small case-control study from North India, with 169 patients, no significant association was identified (*p* = 0.23, yet only 2.3% of controls had migraine and 0% of cases) [40]. Table S3 from Additional File demonstrates a mapping table of each study and the outcomes reported therein.

### Publication bias

Given that none of the analyses in this research exceeded 10 studies, no Egger’s test for publication bias or funnel plots were plotted [42].

**Table 3:** A summary of RVE risks reported in the literature

Author, Year	Risk of any RVE		Risk of RAO		Risk of RVO	
	RR (95% CI)	If adjusted, then adjusted for what?	RR and 95% CI	If adjusted, then adjusted for what?	RR and 95% CI	If adjusted, then adjusted for what?
Ho 2024	1.69 (1.57 to 1.83)	Age, gender, comorbidities, and medications.	**Any reported RAO**: 2.13 (1.84 to 2.48) **CRAO**: 1.37 (1.02 to 1.83) **BRAO**: 1.7 (1.19 to 2.42)	Age, gender, comorbidities, and medications.	**Any reported RVO**: 1.53 (1.4 to 1.68) **CRVO**: 1.4 (1.2 to 1.62) **BRVO**: 1.56 (1.4 to 1.73)	Age, gender, comorbidities, and medications.
Klein 2008	1.71 (1.01 to 2.9)	Age			**CRVO**: 1.1 (0.32 to 3.79) **BRVO**: 1.99 (1.08 to 3.67)	BRVO risk controlled for Age, retinal focal arteriolar narrowing, glaucoma, serum creatinine level, history of barbiturate use, serum phosphorus level, and serum ionized calcium level.
Al-Moujahed 2021	3.48 (3.07 to 3.94)	Age, sex, cardiac diseases (acute coronary syndrome [ACS], valvular disease, heart neoplasm, endocarditis, and rheumatic heart disease), cerebrovascular diseases (stroke, transient ischemic attack, carotid disease, and embolic disease), vascular risk factors (atherosclerosis, hyperlipidemia, hypertension, diabetes mellitus, obesity, tobacco use, and alcohol use), systemic lupus erythematosus (SLE), hematologic diseases (leukemia/lymphoma, homocystinuria, lupus anticoagulant syndrome, antiphospholipid syndrome, protein deficiency anemia, activated protein C resistance, prothrombin gene mutation, and essential [hemorrhagic] thrombocythemia), vasculitis, chronic kidney disease, and retinal vasculitis or inflammation, pregnancy and triptan prescription.	**Any reported RAO**: 3.48 (3.07 to 3.94) **CRAO**: 1.62 (1.15 to 2.28) **BRAO**: 2.09 (1.6 to 2.72)	Age, sex, cardiac diseases (acute coronary syndrome [ACS], valvular disease, heart neoplasm, endocarditis, and rheumatic heart disease), cerebrovascular diseases (stroke, transient ischemic attack, carotid disease, and embolic disease), vascular risk factors (atherosclerosis, hyperlipidemia, hypertension, diabetes mellitus, obesity, tobacco use, and alcohol use), systemic lupus erythematosus (SLE), hematologic diseases (leukemia/lymphoma, homocystinuria, lupus anticoagulant syndrome, antiphospholipid syndrome, protein deficiency anemia, activated protein C resistance, prothrombin gene mutation, and essential [hemorrhagic] thrombocythemia), vasculitis, chronic kidney disease, and retinal vasculitis or inflammation, pregnancy and triptan prescription.		
Gil 2025	1.9 (1.66 to 2.18)	Age, gender, and race.			**Any reported RVO**: 1.9 (1.66 to 2.18)	Age, gender, and race.
Lusk 2024	1.15 (0.79 to 1.67)	Age, Sex, Race/Ethnicity, State, and Co-morbidity adjusted (including cerebrovascular disease, chronic kidney disease, congestive heart failure, coronary artery disease, diabetes mellitus, hyperlipidemia, hypertension, peripheral vascular disease, smoking, and valvular heart disease).	**Any reported RAO**: 1.26 (0.99 to 1.6) **CRAO**: 1.15 (0.79 to 1.67)			
Uppuluri 2021	2.68 (1.88 to 3.69)	Age group (20–29 vs. 30–45), Sex, Ethnicity, Comorbidities, including: Carotid stenosis, Aortic dissection/aneurysm, Atherosclerosis, Congestive heart failure, Diabetes (with & without complications), Hyperlipidemia, Primary hypercoagulable state, Hypertension, Peripheral artery disease, Sickle cell trait, Prior stroke, Syphilis, Systemic vasculitides, Tobacco use, Cardiac valve disease, Glaucoma (including neovascular glaucoma).	**CRAO**: 2.68 (1.88 to 3.69)	Age group (20–29 vs. 30–45), Sex, Ethnicity, Comorbidities, including: Carotid stenosis, Aortic dissection/aneurysm, Atherosclerosis, Congestive heart failure, Diabetes (with & without complications), Hyperlipidemia, Primary hypercoagulable state, Hypertension, Peripheral artery disease, Sickle cell trait, Prior stroke, Syphilis, Systemic vasculitides, Tobacco use, Cardiac valve disease, Glaucoma (including neovascular glaucoma).		
Battagliola 2021	1.523 (0.67 to 3.47)	Not adjusted.			**Any reported RVO**: 1.523 (0.67 to 3.47) **CRVO**: 1.523 (0.67 to 3.47)	Not adjusted.
Tilleul 2011	2.14 (1.13 to 3.53)	Not adjusted.			**Any reported RVO**: 2.14 (1.13 to 3.53) **CRVO**: 1.58 (0.73 to 2.99) **BRVO**: 3.5 (1.79 to 5.37)	Not adjusted.
Nitzan 2026	2.23 (1.6 TO 3.12)	Age at index, sex, race, hypertensive diseases, diabetes mellitus, overweight and obesity, hyperlipidemia, chronic kidney disease, ischemic heart diseases, sleep apnea, anxiety and stress- related disorders, mood disorders, nicotine dependence, and alcohol dependence.				

Abbreviations: **RVE** = retinal vein occlusion, **RAO** = retinal artery occlusion, **RVO** = retinal vein occlusion, **CRAO**: Central Retinal Artery Occlusion, **CRVO**: Central Retinal Vein Occlusion, **BRVO**: Branch Retinal Vein Occlusion

## Discussion

In this meta-analysis, including over 47 million individuals, migraine was associated with an increased risk of RVEs, including both RAO and RVO. Further, evidence from three studies indicates a correlation with maculopathies, though conclusive evidence remains yet to be proven. Based on our findings, the quality of the included studies was generally moderate to high. While the vast majority were large, population-based cohort studies with appropriate exposure ascertainment and adjustment for multiple confounders, substantial heterogeneity lingered across several pooled analyses due to differences in study design and populations. Due to the overabundance of findings from administrative health databases, the possibility of diagnostic misclassification and miscoding in the current state of evidence, and moderate to high-level heterogeneity in calculated effect estimates, a definitive relationship between migraine and RVEs is difficult to be established.

These results align with extensive cerebrovascular literature, where migraine, particularly MwA, has been consistently linked to an increased risk of cerebral ischemic stroke. Large meta-analyses [43, 44] and comprehensive reviews [6, 45] have demonstrated an almost 2-fold increased risk of ischemic stroke among migraineurs, with stronger associations in younger individuals and women. The retina, as an extension of the central nervous system with similar embryologic and microvascular characteristics, may therefore represent another vascular territory susceptible to migraine-related ischemic mechanisms. This is compounded by the vulnerability of the inner retina, which lacks significant collateral supply, causing it to be sensitive to transient hypoperfusion, vasospasm, or embolic phenomena. Our conservative estimate of an RVE relative risk of 1.84 in migraineurs generally conforms with the broader literature on ischemic stroke risk in migraine, where previous meta-analyses suggested twice the risk in migraine (RR in Schurks et al.: 1.74 [46], RR in Spector et al.: 2.04 [43], RR in Etminan et al.: 2.16 [47]). This similarly underscores the shared pathophysiological mechanism between cerebral and retinal infarction, and further strengthens the observed correlation between migraine and RVE.

Evidence from prior meta-analyses (9 studies spanning 675 patients in Ke et al., 2022 [48], 9 studies involving 775 eyes in Pang et al., 2023 [12], and 16 studies with 1620 eyes in Liu et al., 2023 [11]) supports the presence of widespread microvascular dysfunction in migraine, including deep and superficial reductions in the retina’s vascular density. Moreover, retinal imaging studies using OCT and OCT-angiography have demonstrated impaired choroidal perfusion and foveal avascular zone enlargement in migraine patients, even in the absence of overt vascular events [49, 50]. We hypothesize that these microvascular alterations may provide a structural and functional inclination for migraineurs to retinal ischemic events as well as maculopathies.

**Fig. 7:**
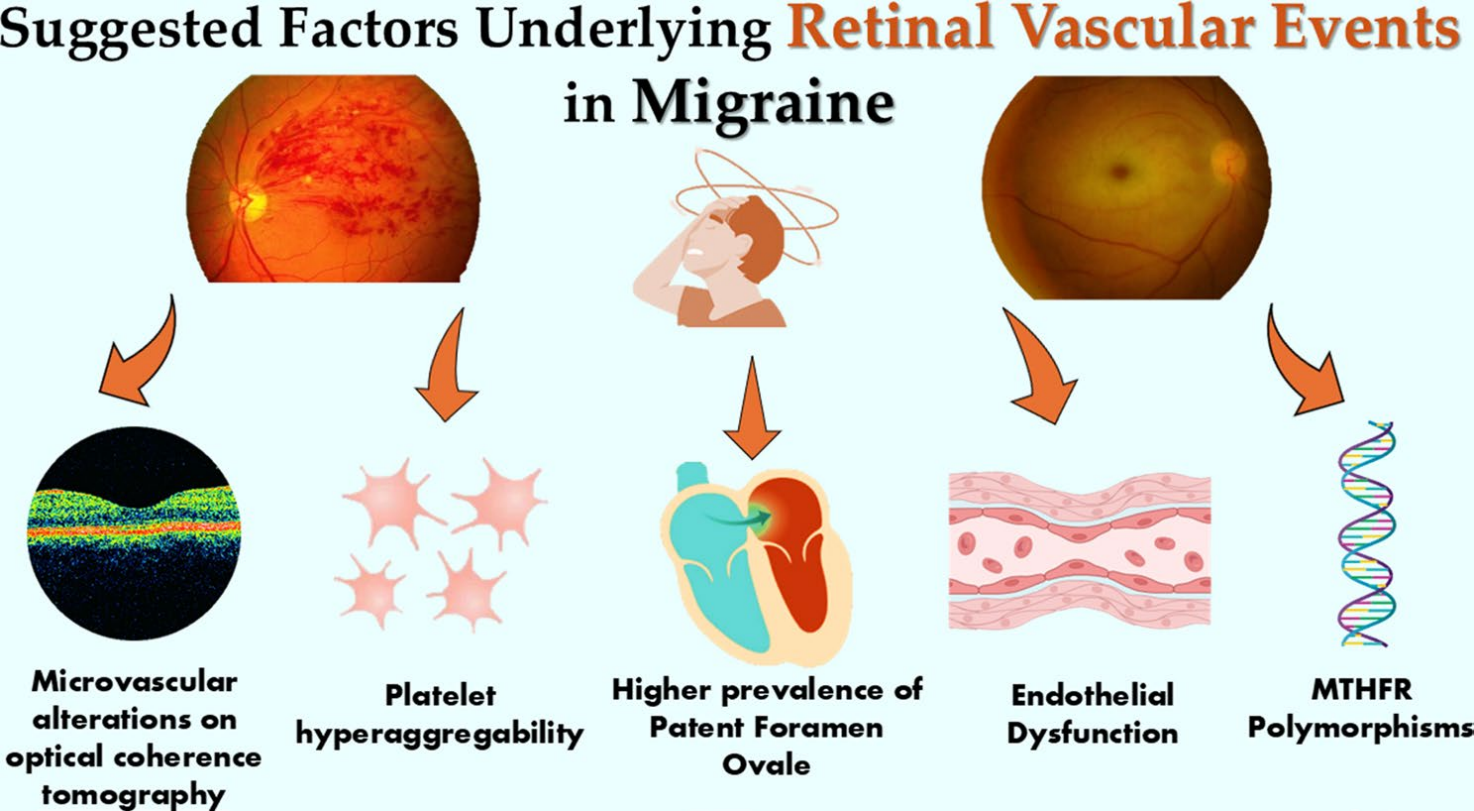
Suggested factors for RVE risk in migraine

Several interacting pathophysiological mechanisms (summarized in Fig. 7) may underlie the associations observed in our meta-analysis. Migraine has been linked to endothelial dysfunction [51, 52], impaired nitric oxide-mediated vasodilation [53], and oxidative stress [54]. Prothrombotic tendencies, including platelet hyperaggregability [55, 56] and increased prevalence of antiphospholipid antibodies, may further increase thrombotic risk [57]. The higher prevalence of patent foramen ovale in MwA raises the possibility of paradoxical embolization to the retinal circulation [58]. Genetic factors such as MTHFR polymorphisms and hyperhomocysteinemia may contribute to endothelial injury [59], while migraine-related vasospasm may directly affect retinal arterioles, particularly during aura phases. Further, while migraine subtypes were not clearly delineated in the included studies, we speculate that those individuals with retinal migraine do stand a theoretically increased risk for RVO and RAO. Retinal migraine is hypothesised to occur due to vasospasms in the retinal vasculature or retinal spreading depression, which can - over time - theoretically increase the risks of overt vascular events [60].

Al-Moujahed et al. [16] provided one of the most comprehensive adjustments for confounding, identifying conditions that increase RAO risk among migraineurs. These included systemic lupus erythematosus, vasculitides, antiphospholipid syndrome, hematologic disorders, and other inflammatory or thrombotic conditions. This identification of significant covariates influencing RAO risk may help identify migraineurs at the greatest risk, as well as provide aid to physicians in targeted screening approaches. Of concern, some analyses in our research yielded a high heterogeneity I^2^ value, which, upon conducting a sensitivity analysis, had the single-source high heterogeneity study omitted. For instance, in our analysis of the risk of any RVE in migraine, the I^2^ statistic was lowered from 94.4% to 55.5% after removing Al-Moujahed et al.^16^, albeit without a change in the direction of the effect. Upon inspecting Al-Moujahed et al.’s [16] methods and population, we discovered how their multivariate analysis controlled for 35 variables (as displayed in Table 3), possibly hinting at a strong independent association of migraine with RVEs. However, after inspecting their results further, we noticed a large difference in reported hazard ratios (95% Cis) among CRAO, BRAO, and other RAO − 1.62 (1.15–2.28), 2.09 (1.60–2.72), and 4.61 (3.94–5.38), respectively. This large effect size observed in other RAO might be due to the misclassification of migraine aura as transient RAO, effectively overestimating the risk of RVEs in migraine reported in Al-Moujahed et al.[16], and contributing to the outlier effect shown in our analysis. Nevertheless, one must keep in mind how the observed heterogeneity is not unexpected in meta-analyses of observational studies, particularly those relying on administrative databases, where variability in coding practices, residual confounding, and outcome misclassification can substantially influence effect estimates.

Age-stratified analyses revealed inconsistent findings across studies, with some reporting higher risks in older individuals and others identifying younger migraineurs as particularly vulnerable. We speculate that this discrepancy might stem from differences in baseline vascular risk, migraine subtype distribution, and comorbidities. Nevertheless, due to the scarcity of data and the inability to conduct subgroup analyses based on age, this speculation cannot be confirmed with certainty. Regarding treatment effects, selected medications, particularly NSAIDs, propranolol, and valproate, were associated with reduced RVE risk in one study only, possibly through anti-inflammatory [61], antithrombotic [62], or endothelial-stabilizing effects [63]. However, we cannot generalize this single-study finding beyond the setting in which it was conducted until future research uncovers the potential protective role of abortive and prophylactic migraine therapies. In a 2026 observational study of over 900,000 individuals, Lusk et al. [34] showed that anti-CGRP therapies did not increase the risk of CRAO, emphasizing their retinal vascular safety, though conclusive evidence is yet to be ascertained. With regard to migraine subtype, our analysis of two studies pointed towards an increased risk of RAO in those with MwA compared to MwoA. This finding conforms with the established literature, where those with aura experience a higher risk of cerebral infarction [64]. In a 2025 proposed risk model for ischemic stroke, McCain et al. developed the MARS+ risk scoring system from a well-characterised cohort, where visual aura contributed 2 points, similar to important risk factors such as hypertension, body mass index ≥ 30, and atrial fibrillation [65]. Hence, we believe those with MwA need extra access to care and scrutiny for retinal ophthalmological emergencies.

In an interesting large-scale meta-analysis of over 300,000 individuals, Wang et al. compared RAO cases with controls and revealed a 264% increased risk of stroke in RAO, especially during the initial months following RAO [66]. Our research, in turn, highlights migraine as a positive influencing variable towards RAO; taken together, and given the established link between migraine and stroke, we recommend a proactive approach in which migraineurs with RAO have comprehensive follow-up and screening during the RAO hospitalization period and beyond. Further, in case of pre-existing risk factors like diabetes mellitus, smoking, or blood clotting tendencies, we suggest that ophthalmologists and neurologists conduct a comprehensive vascular evaluation and possibly prescribe antiplatelet or anticoagulant therapies to reduce another cerebrovascular insult following RAO.

Beyond occlusive events, migraine was associated with several maculopathies. Kuang et al. [33] demonstrated an increased risk of neovascular AMD, while Nitzan et al. [32] reported a strong association with CSCR. In addition, several case reports [67–69] have described PAMM in migraine patients, supporting the role of deep retinal capillary plexus ischemia. The pathogenesis of neovascular AMD - while not fully understood - is believed to involve vascular endothelial growth factors (VEGF), which are inflammatory factors that increase vascular permeability [70, 71]. Migraine’s pathogenesis may overlap with neovascular AMD, given evidence of VEGF dysregulation and the triggering of inflammatory signaling pathways during the attack [72, 73], providing some explanation for the correlation seen in Kuang et al.’s [33] nationwide analysis. With regard to CSCR, similar mechanisms are shared between migraine and CSCR, such as stress-related dysregulated vascular tone [37]. Importantly, CSCR symptoms, like metamorphopsia or dyschromatopsia, might be misdiagnosed by neurologists as the visual aura of migraine. This link, though only in two studies, between CSCR and migraine, acts as a reminder for headache specialists to consider CSCR as a differential diagnosis before definitively diagnosing aura. Overall, these conclusions expand the clinical spectrum of migraine-related retinal pathology beyond vascular occlusion.

### Strengths and limitations

To the best of our knowledge, this endeavor represents the first systematic review and meta-analysis examining migraine as an associated risk factor with retinal vascular events and maculopathies. The inclusion of multiple databases, in addition to searching for relevant conference abstracts and placing no restrictions on linguistics or publication period, ensures a comprehensive approach to the literature. This research also extended to capturing and analyzing further important outcomes, such as risks of central/branch forms of RAO and RVO, the impact of covariates such as medication use and migraine subtype, among others.

Nevertheless, a number of limitations are worth mentioning: Most included studies were retrospective, limiting causal inference. Although we conducted dedicated analyses using adjusted models and the best adjusted RRs, residual confounding (socioeconomic status, physical activity, or others) remains conceivable. Case-control studies are also subject to recall bias, complicating correlations between variables. Diagnostic misclassification of migraine subtype and retinal outcomes is possible, particularly in administrative databases. Further, due to the symptomatic overlap, patients diagnosed with transient RAO might have been experiencing visual aura instead, possibly leading to an overinflation of RAO cases in the migraine group. In some of our analyses, a high heterogeneity I^2^ statistic value was calculated; nevertheless, sensitivity analyses identified single-study sources of heterogeneity – possibly due to population differences at baseline or overestimation of the risk due to misclassification error inherent to administrative health database studies. Generalizability to Africans, South Americans, or Middle Easterners may be limited due to the abundance of evidence from North America, Europe, and East Asia. Significant heterogeneity was observed across pooled analyses due to methodological and population-level differences. It is worth mentioning that some studies did not control for medication use in migraine. Though a single study disproved this, it is possible that triptan use raised RVE risk through its vasoconstrictive properties. Finally, data on migraine severity, duration, and attack frequency were unavailable in multiple studies.

### Recommendations for future studies

Prospective studies are needed to clarify the temporal relationship between migraine and RVEs and to explore dose-response effects on migraine frequency and severity. Further investigation into the impact of migraine medications, particularly long-term CGRP inhibition, is needed. Dedicated pharmacoepidemiologic studies are necessary to disentangle medication effects from disease-related risk. Moreover, we believe the identification of clinical characteristics or co-morbidities that may influence the linkage between migraine and RVEs is vital, as it can help in tailored prevention strategies and a stratification of future RVE risk. In addition to studies integrating retinal imaging, genetic profiling, and inflammatory biomarkers may help identify high-risk subgroups and inform targeted prevention strategies.

## Conclusions

In this largest-to-date systematic review and meta-analysis of observational studies, encompassing over 47 million individuals, migraine was associated with a significantly increased risk of RVEs, including both retinal arterial and venous occlusions. The association persisted across adjusted analyses and was particularly notable for BRAO and BRVO. Yet, a definitive link between migraine and RVEs is difficult to establish, given constraints from miscoding/misclassification risk in administrative health databases. In addition, moderate-level heterogeneity in the pooled analysis for all RVO events in migraine suggests that baseline differences in population characteristics or follow-up durations may complicate establishing an independent link. Finally, evidence also suggests a potential relationship between migraine and selected maculopathies, including neovascular AMD and CSCR, although data remain limited.

Clinically, our results underscore the importance of interdisciplinary awareness as well as support proactive ocular monitoring. Ophthalmologists should consider migraine history when evaluating patients at risk for RVEs, while neurologists and primary care physicians should remain vigilant for visual symptoms suggestive of retinal pathology rather than attributing them solely to aura. Given the association between RAO and subsequent ischemic stroke, migraineurs presenting with RAO may warrant comprehensive vascular evaluation and close follow-up to mitigate future cerebrovascular risk.

Although relative risks were elevated, the absolute event rate remains low, and clinical decisions should evaluate the baseline vascular risk. Taken together, in observational-based evidence, migraine appears to be associated with retinal vascular events, reinforcing its role as a systemic vascular condition rather than an isolated headache disorder.

## Supplementary Information

Below is the link to the electronic supplementary material.

Supplementary Material 1

## Data Availability

The dataset(s) supporting the conclusions of this article is(are) included within the article (and its additional file(s)).

## Abbreviations

MwA: Migraine with Aura
RVE: Retinal Vascular Events
RAO: Retinal Artery Occlusion
RVO: Retinal Vein Occlusion
CRAO: Central Retinal Artery Occlusion
CRVO: Central Retinal Vein Occlusion
BRAO: Branch Retinal Artery Occlusion
BRVO: Branch Retinal Vein Occlusion
AMD: Age-related Macular Degeneration
CSCR: Central Serous Chorioretinopathy
MwoA: Migraine Without Aura
RR: Risk Ratio
CGRP: Calcitonin Gene-Related Peptide
CI: Confidence Interval
PAMM: Paracentral Acute Middle Maculopathy
PRISMA: Preferred Reporting Items for Systematic Reviews and Meta-analyses
