# Supplementary material for: Migraine as a risk factor for retinal vascular events and maculopathies: a systematic review and meta-analysis of 47 million individuals
Source: J Headache Pain. 2026 Apr 15;27(1):107. doi: 10.1186/s10194-026-02353-8 (PMC13085481; doi:10.1186/s10194-026-02353-8)
Supplement: Supplementary file 1 — Supplementary Material 1 [file 10194_2026_2353_MOESM1_ESM.docx]

**Search terms:**

(Migrain*) **AND** ("retinal stroke*" OR "retinal artery occlusion*" OR "retinal arterial occlusion*" OR "retinal vascular occlusion*" OR "retinal vessel occlusion*" OR "retinal ischem*" OR "retinal infarc*" OR "retinal vein occlusion*" OR "retinal venous occlusion*" OR "retinal vein thromb*" OR "retinal venous thromb*" OR "branch vein occlusion*" OR "branch venous occlusion*" OR "branch vein thromb*" OR "branch venous thromb*" OR "amaurosis fugax" OR "Transient monocular visual loss" OR "Transient monocular vision loss" OR "transient vision loss" OR "transient visual loss" OR "macul*")

**
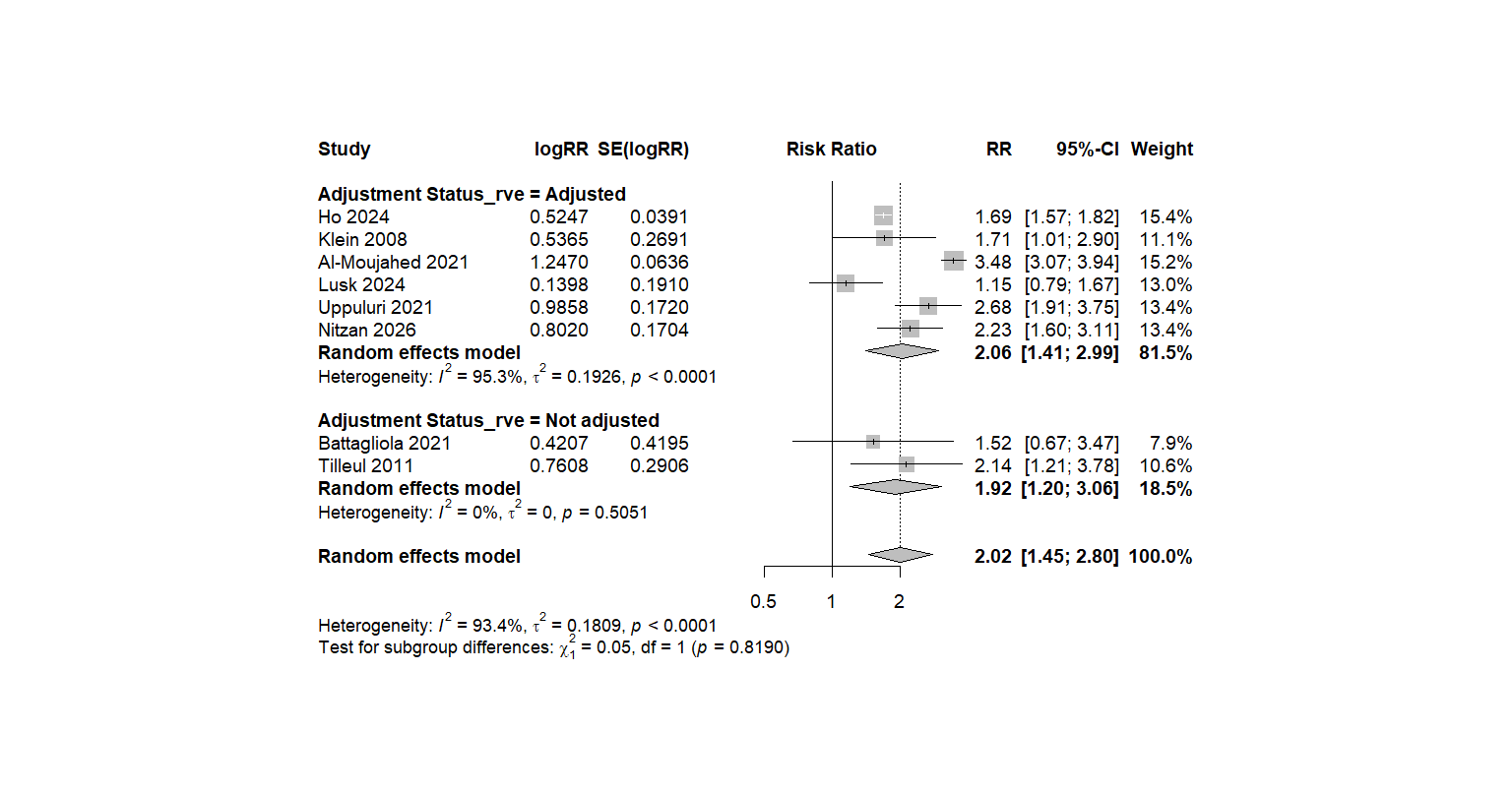
**

**Figure S1** - Risk of any RVE in individuals with migraine versus without migraine (after removing Gil et al.)


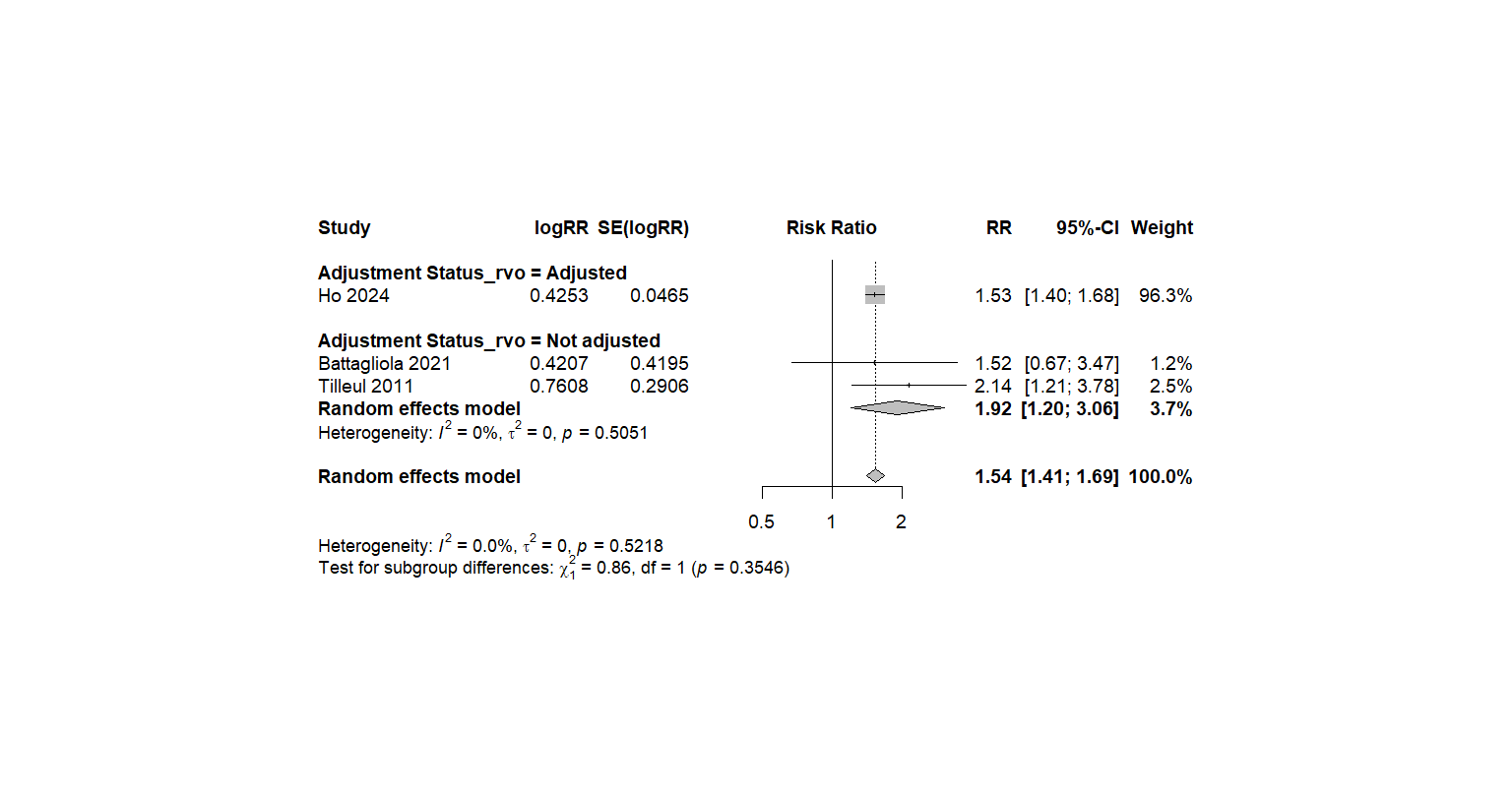


**Figure S2** - Risk of RVO in individuals with migraine versus without migraine (after removing Gil et al.)


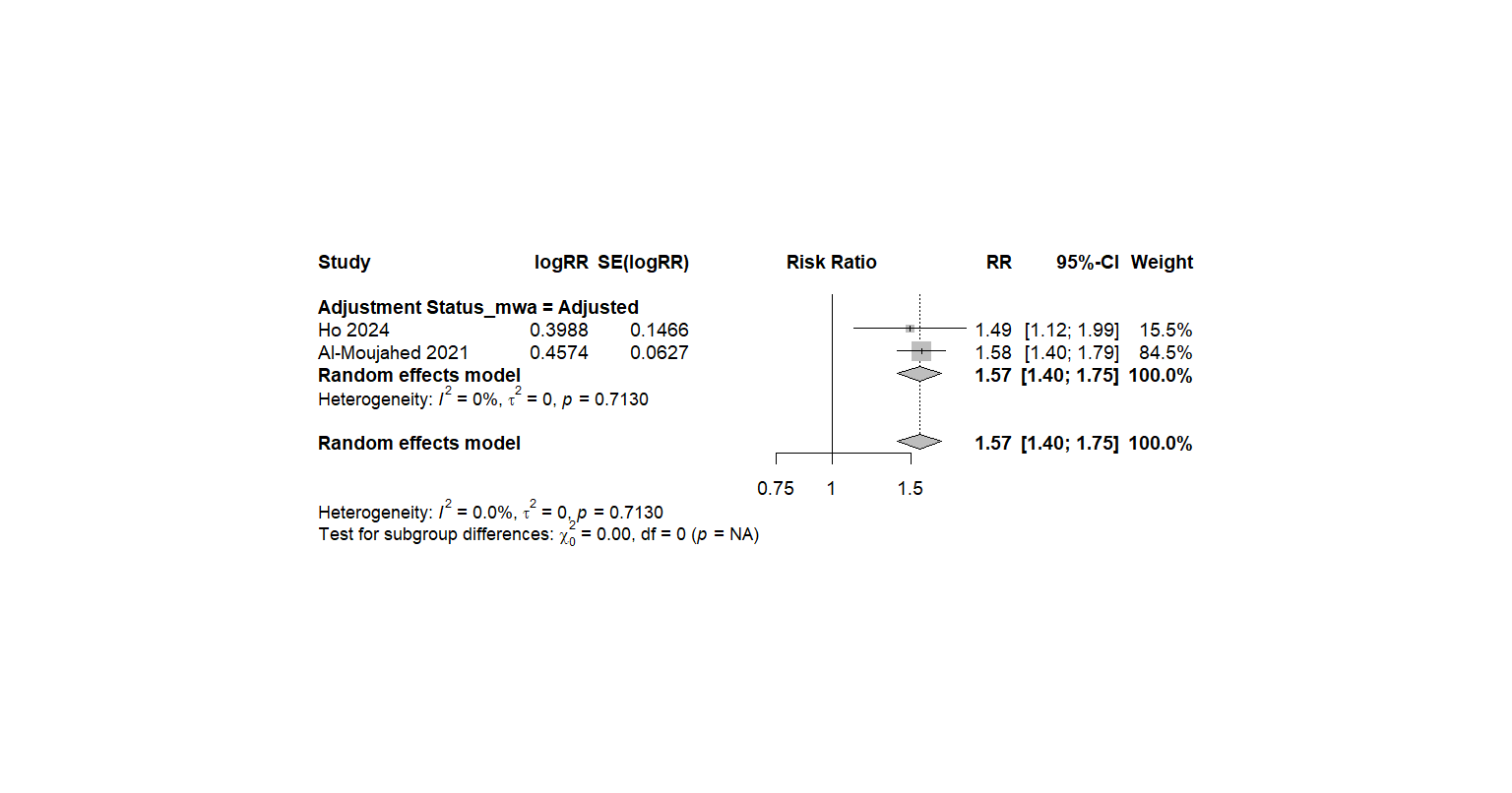


**Figure S3** - Risk of RAO in individuals with MwA versus those with MwoA

**Table S1: Studies Excluded by Full-text Screening**

| **Study** | **Year of publication** | **DOI** | **Exclusion Reason** |
| --- | --- | --- | --- |
| Risikofaktoren bei Patienten mit Amaurosis fugax  [Risk Factors in Patients with Amaurosis Fugax] | 1996 | 10.1055/s-2008-1035187 | Wrong Outcomes |
| MARS: Migraine and Retinal Stroke - A Population-Based Cohort Study of 39 Million Patients | 2024 | https://dx.doi.org/10.1161/str.55.suppl_1.WP252 | Abstract later published as full-text |
| Retinal artery occlusion in young patients without typical cardiovascular risk factors: etiologies, prognosis, and suggested work-up. | 2024 | https://dx.doi.org/10.1007/s00417-024-06527-5 | Wrong Outcomes |

**Table S2: Effect measures reported in each included study**

| **Study** | **Effect Measure** |
| --- | --- |
| Al-Moujahed 2021 | HR |
| Battagliola 2021 | OR |
| Gil 2025 | RR |
| Ho 2024 | HR |
| Klein 2008 | OR |
| Kuang 2022 | OR |
| Lusk 2026 | HR |
| Lusk 2024 | HR |
| Nitzan 2026 | HR |
| Tilleul 2011 | Raw percentage proportion of cases and control |
| Uppuluri 2021 | OR |
| Mansour 2017 | Raw percentage proportion of cases and control |
| Kumawat 2021 | OR |

**Table S3: Mapping of each study and its reported outcomes**

| **Study** | **Outcome Reported?** | | | | | | | | | **Notes** |
| --- | --- | --- | --- | --- | --- | --- | --- | --- | --- | --- |
|  | **Any RVE** | **Any RAO** | **CRAO** | **BRAO** | **Any RVO** | **CRVO** | **BRVO** | **CSCR** | **AMD** |  |
| Al-Moujahed 2021 | Yes | Yes | Yes | Yes | - | - | - | - | - |  |
| Battagliola 2021 | Yes | - | - | - | Yes | Yes | - | - | - |  |
| Gil 2025 | Yes | - | - | - | Yes | - | - | - | - |  |
| Ho 2024 | Yes | Yes | Yes | Yes | Yes | Yes | Yes | - | - |  |
| Klein 2008 | Yes | - | - | - | - | Yes | Yes | - | - |  |
| Kuang 2022 | - | - | - | - | - | - | - | - | Yes |  |
| Lusk 2024 | Yes | Yes | Yes | - | - | - | - | - | - |  |
| Tilleul 2011 | Yes | - | - | - | Yes | Yes | Yes | - | - |  |
| Uppuluri 2021 | Yes | - | Yes | - | - | - | - | - | - |  |
| Lusk 2026 | - | - | - | - | - | - | - | - | - | Lusk 2026 reports on the risk of CRAO in individuals using anti-CGRP therapies. |
| Nitzan 2026 | Yes | - | - | - | - | - | - | Yes | - |  |
| Mansour 2017 | - | - | - | - | - | - | - | Yes | - |  |
| Kumawat 2021 | - | - | - | - | - | - | - | Yes | - |  |
